# An expressed, endogenous Nodavirus-like element captured by a retrotransposon in the genome of the plant parasitic nematode *Bursaphelenchus xylophilus*

**DOI:** 10.1038/srep39749

**Published:** 2016-12-22

**Authors:** James A. Cotton, Sascha Steinbiss, Toshiro Yokoi, Isheng J. Tsai, Taisei Kikuchi

**Affiliations:** 1 Wellcome Trust Sanger Institute, Wellcome Genome Campus, Hinxton, Cambridge, CB10 1SA, UK; 2 Forestry and Forest Products Research Institute, Tsukuba 305-8687, Japan; 3 Division of Parasitology, Faculty of Medicine, University of Miyazaki, Miyazaki 889-1692, Japan; 4 Biodiversity Research Center, Academia Sinica, Taipei 11529, Taiwan

## Abstract

Recently, nematode viruses infecting *Caenorhabditis elegans* have been reported from the family Nodaviridae, the first nematode viruses described. Here, we report the observation of a novel endogenous viral element (EVE) in the genome of *Bursaphelenchus xylophilus*, a plant parasitic nematode unrelated to other nematodes from which viruses have been characterised. This element derives from a different clade of nodaviruses to the previously reported nematode viruses. This represents the first endogenous nodavirus sequence, the first nematode endogenous viral element, and significantly extends our knowledge of the potential diversity of the *Nodaviridae*. A search for endogenous elements related to the Nodaviridae did not reveal any elements in other available nematode genomes. Further surveillance for endogenous viral elements is warranted as our knowledge of nematode genome diversity, and in particular of free-living nematodes, expands.

The first evidence that nematodes might host viruses were a number of reports of virus-like particles by electron microscopy (see Bird and Bird^1^ pp276–280 for a review of this early literature^2^). This work suggested that a number of free-living and plant parasitic nematode species could have viral infections^3^, but most early reports did not further characterise the viruses themselves, although Poinar and Hess^4^ identified a virus related to the iridoviruses in the cytoplasm of *Romanomermis culcivorax*. The recent discovery of three nematode nodaviruses^5^,^6^ from natural populations of *Caenorhabditis elegans* and *C. briggsae*, represented the first viruses known to naturally infect free-living nematodes and has rekindled interest in nematode virology.

Nodaviruses are positive sense single-stranded RNA viruses. This group was previously known to infect both vertebrate (fish) and invertebrate (arthropod) hosts, and the classical view of this family divides them into two clades (alpha- and beta-Nodaviridae) representing these divergent host groups. The nematode viruses thus significantly expanded the known host range of nodaviruses, and expanded the known diversity of the viruses themselves: the *Caenorhabditis* viruses form a group only distantly related to the arthropod nodaviruses. The isolation of viruses infecting the model nematode permits the investigation of nematode-virus interactions using a natural infection^6^,^7^. Research on these viruses has demonstrated that they infect intestinal cells in the worm^7^, have revealed a strikingly different capsid structure of Orsay (one of the *C. elegans* viruses) to the arthropod betanodaviruses^8^ and is starting to investigate the molecular biology^9^ and even develop approaches for genetic manipulation^10^ of this virus.

Following this discovery, a number of other potential nematode viruses have been described, significantly expanding the diversity of both nematode hosts and of the viruses infecting them. Four negative sense RNA viruses belonging to different families have been described in transcriptomic data from the plant parasitic nematode *Heterodera glycines*^11^. A survey of nematode EST data also revealed sequences related to the positive sense single-stranded RNA Picornavirales in two plant nematodes, *Globodera pallida* and *Heterodera schachtii*^12^. While the nature of these data, and while the presence of nematode-vectored plant viruses within the picornavirales^13^ make it impossible to be certain whether these are nematode viruses or contaminants from the plant material, it seems likely that these represent another novel group of nematode viruses.

While these findings have given us a glimpse of the possible diversity of nematode viruses, it seems likely that these rather sporadic and opportunistic findings may significantly underrepresent both the host range and diversity of viruses themselves: as far as we are aware, only two studies^11^,^12^ have systematically searched for virus-like sequence in a nematode, and these only examined transcriptomic data for RNA viruses. In other taxonomic groups, mining genomic data to find endogenous viral elements – DNA sequences derived from viruses following integration into the genome – has revealed a widespread and diverse menagerie of endogenised viral sequences^14^. These endogenous viral copies are, of course, particularly widespread for retroviruses where integration into the host genome is a obligate part of the lifecycle^15^ but integrated elements derive from a wide range of viruses, including viruses that have an exclusively RNA lifecycle. While the first report of endogenous elements derived from RNA viruses was only in 2004^16^, there have subsequently been reports of at least 10 families in animal genomes^14^,^17^, including humans^18^ and other reports in fungi and bacteria (see Holmes *et al*.^17^ for review). Study of ‘fossil’ copies of viruses has given valuable insights into the evolution of both viruses^19^,^20^,^21^ and hosts^22^,^23^.

Here, we exploit the published sequence data for *Caenorhabditis* nodaviruses and published genomic data for nematodes to investigate the potential diversity of integrated nodavirus-derived elements in nematodes. We find an endogenous nodavirus-related sequence in the genome of *Bursaphelenchus xylophilus*^24^, the pine wood nematode: a plant parasitic nematode that causes severe damage to forestry and forest ecosystems^25^. The nodavirus-related sequence is embedded in a degerate LTR retrotransposon, suggesting a route via which this positive sense RNA virus could have become integrated into a nematode genome.

## Results

### Discovery of eBxnv-1

**Figure 1:**
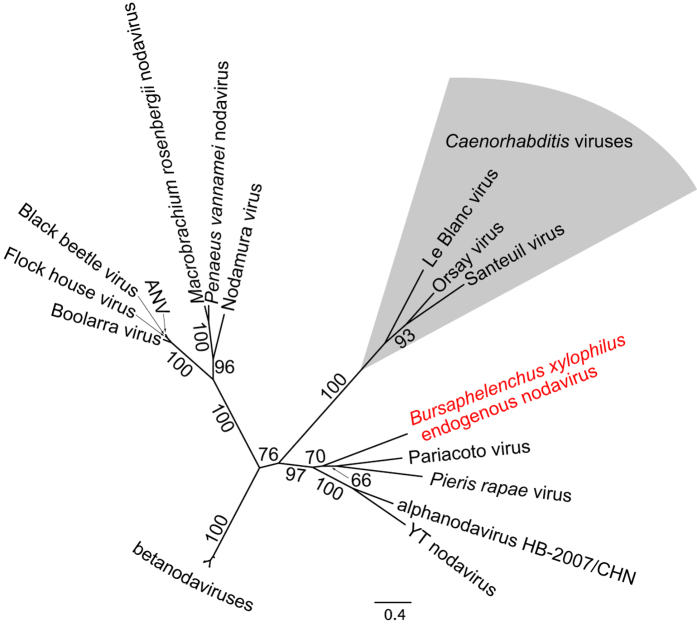
Phylogeny of eBxnv-1 with RNA-dependent RNA polymerase genes of other nodaviridae. Values next to branches are bootstrap proportions for the partition implied by that branch, based on 1000 replicates. Bootstrap values for relationships within the beta-nodaviruses and between ANV (*Drosophila melanogaster* Amercan nodavirus), Flock house and Black beetle viruses are not shown.

The search strategy we employed found only a single blastp hit with E-value < 10^−2^, in the genome of *Bursaphelenchus xlyophilus*^24^ to BUX.s01281.241 for all three RNA-dependent RNA polymerase sequences, with blastp E-values between 2 × 10^−18^ and 2 × 10^−25^ (bit scores between 91 and 113). The predicted gene model in *B. xylophilus* is a single exon gene encoding a hypothetical protein of 570 AA, with similarity to Prosite model PS5057 (*RNA-dependent RNA polymerase of positive ssRNA viruses catalytic domain*, at residues 407–529)^26^, and to the Superfamily HMM SSF56672 *(DNA/RNA polymerases superfamily*, at residues 407–529)^27^. A search using NCBI blast for sequence similar to this protein in the Genbank nr database found significant similarity only to RNA-dependent RNA polymerases of nodaviruses (Supplementary Table S3c). Tblastn identified only the same similarity regions as blastp, but blastn searches produced multiple small (around 60 bp) but significant hits for a region of Le Blanc virus to a number of *Caenorhabditis* species (4 hits in *C. brenneri*; 1 hit in *C. briggsae*; 61 hits in *C. japonicum*; 2 in *C. remanei*; 1 in *C. sp 5* and 4 in *C. sp 11*). These sequences apparently form part of the internal transcribed spacer (ITS1) of the rRNA array in these species, and could represent capture of some host RNA by this virus, but the biological significance of these matches is unclear. Phylogenetic analysis confirms that the *Bursaphelenchus* predicted protein is related to nodavirus RDRP (Fig. 1). Intriguingly, there is strong support for this protein to be closely related to a subgroup of the arthropod-infecting alphanodavirus from Lepidoptera. The endogenous nodavirus we have identified – provisionally called eBxnv-1 – is thus only distantly related to the clade of *Caenorhabditis* nodaviruses.

### Structure of eBxnv-1

The gene model BUX.s01281.241 is significantly shorter than the polymerase proteins for other nodaviruses, at only 570 amino acid residues versus between 950 and 1050 for known virus proteins. In general, the amino acid sequence of these viruses is rather poorly conserved, and the eBxnv-1 is particularly divergent. Intriguingly, however, three conserved domains characteristic of nucleic acid polymerases^28^ and conserved in virus RNA-dependent RNA polymerases^29^ are clearly present in eBxnv-1 and align well against those in other nodavirus sequecnes (Supplementary Fig. S1; see PROSITE PS50507). In particular, the putatively catalytic Asp residues in domains IV and VI and Asn residue in domain V are present.

**Figure 2:**
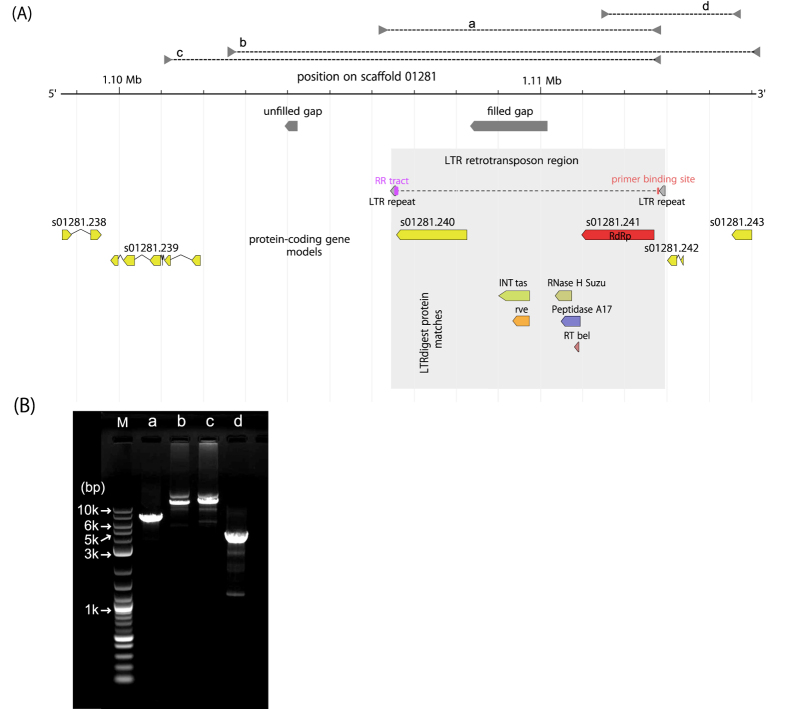
(**A**)Structure of the putative endogenised nodavirus RNA1 locus in the *Bursaphelenchus xylophilus* genome, the surrounding LTR retrotransposon and adjacent gene models. (**B**) Genomic PCR confirming assembly correctness as this locus, and so confirming endogenous nature of this virus-derived gene. Lower case alphabets (a–d) on each lane represent amplified genome regions shown in (**A**). M; 2-log ladder DNA size maker.

With any draft genome assembly, individual scaffolds could represent contamination rather than genuine sequence from the target organism. To confirm that the identified gene model is part of the *B. xylophilus* genome, we checked that the adjacent gene models show similarity to nematode proteins. While both predicted proteins 5′ to the eBxnv-1 locus show significant similarity to proteins from other nematodes, none of the three 3′ adjacent proteins have convincing BLAST hits to any proteins in the NCBI database (Supplementary Table S3a,b,d–f). Furthermore, a gap region between BUX.s01281.240 and BUX.s01281.241 makes the co-linearity of these genes in the genome assembly uncertain. We performed genomic PCR to fill this gap, with direct sequencing of the PCR products revealing the gap was of 1,828 bp. This genomic PCR confirms the correct assembly of this region, linking the identified endogenous sequence to these adjacent genes and to the rest of the scaffold (Fig. 2). Further investigation of the scaffold after filling this gap revealed the region has long terminal repeat (LTR) transposon-like structure (Fig. 2) with two LTRs, polypurine tract, primer binding site and *pol* coding sequences including peptidase and integrase between the two LTRs, but no sign of the *gag* proteins found in most full-length LTR transposons. This LTR appears to be a member of the Bel/Pao retroelements, and particularly to be related to members of the Tas clade of this group (Supplementary Table S3g,h), which includes elements described from both cnidarians and nematodes including *Caenorhabditis elegans, Brugia malayi* and *Trichinella spiralis*^30^. The predicted protein sequence show similarity to both the ribonuclease H and reverse transcriptase domains of the Pol open reading frame of these other elements, but are either only distantly related or degenerate. The LTR sequences themselves show complete conservation over 132 nucleotides and no homology outside this region – the Tas element has LTRs of around 250 nt, and the LTRs of all other elements in this clade are all longer. The RdRp-like protein is located within this LTR structure, along with an additional protein of unknown function (Fig. 2).

### Expression of eBxnv-1-containing element

To investigate expression, RNA-seq reads were mapped to the gap-filled reference genome. The 100 bp-paired RNA-seq were mapped throughout the region between the two LTRs, but mapping coverage is as low as ~20x (FPKM ~60) (Supplementary Fig. S2A) and no significant expression difference between developmental stages (propagative-stages and dauer-stage) was observed. De novo assembly of the RNA-seq using Trinity generated a single contig of 7088 bp that again covers the entire LTR region. In addition, RT-PCR using mixed-stage nematodes detected as long as ~2 kb and ~5 kb fragments (Supplementary Fig. S2B). These results suggest this region is expressed at a low level as a single long transcript. Stage specific expression was further examined using RT-qPCR. We observed similar expression levels in adult females, males, L2 and L3/L4 although the expression levels are low compared to the actin gene (approx. 1/1000 of the level) used as an endogenous control in the qPCR experiment (Supplementary Fig. S2C). No virus particles were observed in a nematode homogenate under the electron microscope (Supplementary Fig. S3).

### Other repeat elements and other copies of eBxnv-1 in the Bursaphelenchus genomes

A genome-wide, structure-based *de novo* search for LTR retrotransposons identified 1,301 potential candidate elements, including the element around eBxnv-1. 88 of those showed at least one HMM hit to a selection of endogenous retrovirus-related protein domain models from Pfam and GyDB. Only four show a set of domains which could be considered complete for transposition (RT, protease, integrase and RNaseH). Comparison of the eBxnv-1-containing element with the other candidates resulted only in short and low-specificity hits to 11 predicted candidates, suggesting they are not additional copies of the element in question. Genome-wide similarity searches for the eBxnv-1-containing element only delivered 9 insertion fragments (some on short contigs) and a number of isolated LTRs.

**Figure 3:**
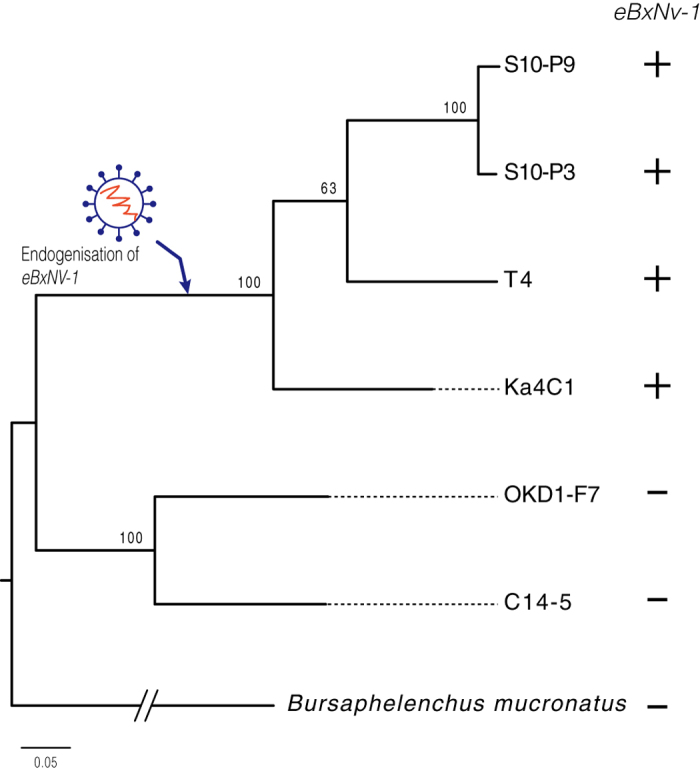
Endogenisation of eBxnv-1 in *B. xylophilus.* A maximum likelihood phylogenic tree for six *B. xylophilus* strains and the related species *B. mucronatus* based on a total of 1121 SNP sites. *B. mucronatus* was used as an outgroup. Plus signs indicate the presence of the eBxnv-1 element in that strain was identified by genomic sequence reads and PCR, minus indicates failure to detect the element. The arrow indicates the inferred timing of the integration of eBxnv-1 in *B. xylophilus*.

Next, we examined presence/absence of the eBxnv-1 element in other strains of *B. xylophilus* and in its closely related species by mapping of gDNA Illumina reads and PCR amplification using the specific primers (Supplementary Table S1). The element was detected in four strains of *B. xylophilus* (S10-P3, S10-P9, T4 and the reference strain Ka4C1) at the same genome location, but not in two other *B. xylophilus* strains (OKD1-F7 and C14–5) or *B. mucronatus* (Fig. 3), suggesting eBxnv-1-containing element were endogenised after the speciation of *B. xylophilus* and *B. mucronatus*. No SNPs and indels were detected in the area of eBxnv-1 (a total of 1713 bp) in the four *B. xylophilus* strains although their genomes are shown to be highly diverged^31^.

## Discussion

Nodaviruses are small, non-enveloped +sense ssRNA viruses, with RNA1 encoding the RNA-dependent RNA-polymerase and protein B2 (ecoded from the 3′ end of RNA1), and RNA2 encoding the capsid protein precursor^32^,^33^. Our results suggest that the RNA1 transcript of a nematode-infectious nodavirus infecting the germ line of *B. xylophilus* has been reverse transcribed by a cellular reverse transcriptase activity, captured by non-homologous recombination by an LTR retroelement and inserted into the host genome. This is the first evidence of a nematode nodavirus outside *Caenorhabditis spp*., expanding the known host-range of nodaviridae; the ancestral virus our element descended from represents a distinct lineage from the *Caenorhabditis* viruses or any other nodavirus. The Orsay virus apparently causes damage to intestinal cells in *C. elegans*^6^, so if an active nodavirus infective *Bursaphelenchus* was isolated, and shown to be pathogenic, this could represent a possible avenue for biological control of pine wilt disease^34^.

Our results are – to the best of our knowledge - the first confirmation of an endogenous viral element in a nematode genome, although the virus-related transcripts previously identified^11^,^12^ could be from endogenous loci. This builds on the growing evidence of extensive endogenised virus elements found by in-silico screening of mammal, bird and insect genomes against a library of non-retroviral peptides using methodology similar to that employed here^14^. As commonly found in EVEs in other systems^14^,^21^, the endogenous copy we identify is not closely related to any of the exogenous viruses within this family, and the most closely related viruses use insect, not nematode, hosts. This finding thus expands our knowledge of the diversity of *nodaviridae* and of the diversity of host-virus relationships in this group. In common with other recently discovered EVEs related to ssRNA^+^ viruses, eBxnv-1 appears to be present in very low copy number – in this case, a single copy – perhaps due to low viral mRNA copy number^17^.

The entire LTR element – including the nodavirus-derived ORF – is expressed at a low level across all *B. xylophilus* life history stages investigated. Expression of the endogenised viral element, and the conservation of several key domains required for polymerase function in an otherwise divergent amino acid sequence, raises the possibility that this gene is functional within nematode cells. A different virus RdRp gene has recently been propose to be functioning in some bat genomes^35^ – while it is hard to be sure about possible functions, these authors speculate that this mammalian protein could play a role in either anti-viral defense or in the RNAi pathway. While rare, a number of examples are known where viral proteins have been ‘domesticated’ by the cellular host, particularly being recruited to anti-viral defence. The most well-explored examples are in mammals (see ref. 23 for review) but this process is likely to be more widespread (e.g. ref. 36). The fact that the only appearance of this LT element in the present-day *B. xylophilus* genome is the copy enclosing eBxnv-1 makes is tempting to speculate that there could be some functional explanation for the persistence of this locus, but we have no evidence for any such function.

The presence of a +sense RNA virus integrated into the *Bursaphelenchus* genome implies that these nodaviruses can infect germline cells and that genetic material derived from these viruses can find its way into the nucleus. As the nodavirus has no reverse transcriptase activity, the ancestral viral RNA of our eBxnv-1 element must have interacted with an exogenous protein with reverse transcriptase activity to generate a DNA molecule^14^,^17^,^18^. In this case, we presume that this RT was from the LTR element we now find surrounding the eBxnv-1 protein itself and speculate that the recombination between the retroelement and nodavirus templates occurred during the reverse transcription process itself by RT copy-choice recombination, and that this recombinant molecule could then integrate into the host DNA. This process of retroelement capture of a non-retrovirus material has been experimentally demonstrated in a mammalian system^37^, but we are aware of only one previous natural example^38^, and in that case only the terminal repeats of the implicated element remained.

The LTR retrotransposon we identify in the *B. xylophilus* genome is clearly derived from an element related to Bel/Pao elements previously identified in other nematodes. The long terminal repeat sequences themselves appear to be truncated but a well-conserved region is still present. However, because the protein-coding open reading frames appear to be highly degenerate, the LTR element is certainly non-functional. The element appears to be restricted to one clade of *B. xylophilus* strains and absent in the related species *B. mucronatus,* suggesting that the endogenisation event occurred relatively recently in *B. xylophilus*. It is noteworthy that the endogenised region in the element (i.e. eBxnv-1 gene) retains key catalytic sites and is highly conserved in the four *B. xylophilus* strains. Taken together with the fact that the region is expressed in multiple developmental stages of the nematode, albeit weakly, suggests that the RdRP gene could be functional although the evolutionary significance for the host is unclear. Similar expression and possible exaptation of endogenised RdRp has been observed in *Eptesicus* bats^18^.

## Methods

BLAST searches (blastp, tblastn and blastn) with the nucleotide and predicted protein sequences for all 3 ** nodavirus genomes available against the genomes and predicted proteomes for all 15 nematode genomes available in WormBase release WS233^39^ (7 *Caenorhabditis* spp., *Ascaris suum, Brugia malayi, Bursaphelenchus xylophilus, Haemonchus contortus, Meloidogyne hapla, Pristionchus pacificus, Strongyloides ratti and Trichinella spiralis,* with genomic sequence only (no predicted proteins) available for *Heterorhabditis bacteriophora* and *Meloidogyne incognita*). Protein sequences for the RNA-dependent RNA polymerase protein of other known nodaviruses were downloaded from Genbank (see Supplementary Table S1), and aligned with mafft v6.857^40^ with the ‘—auto’ option. The alignment was then cleaned with trimAl v1.4 using the ‘-automated1’ option^41^. Phylogenetic analysis was performed using RAxML v.7.2.8^42^, using a model of amino-acid substation (LG+F) chosen as that minimizing AICc and a discretized gamma distribution to model rate variation across sites plus an estimated proportion of invariant sites. Inference used the rapid hill-climbing algorithm of RAxML.

An assembly gap between BUX.gene.s01281.240 and BUX.gene.s01281.241 was filled by PCR direct sequencing. PCR amplification was performed using Tks Gflex DNA Polymerase (Takara, Japan) as recommended by the manufacturers; 30 cycles of PCR (in 30 μl volume) were performed with 10 sec at 98 °C and 7 min at 68 °C with 2 min of 94 °C initial denaturation. PCR products were sequenced on ABI Prism 3130 DNA sequencer using BigDye Terminator v3.1 (Applied Biosystems). The primers used for PCR and sequencing are listed in Supplemental Table S2. RNA-seq reads were obtained from poly-A selected RNA extracted from mixed-stage and dauer-stage *B. xylophilus*^43^ using Illumina HiSeq2000 platform and standard protocols (http://www.illumina.com/). Reads were mapped against the reference genome using TopHat software, v 2.0.6 (–mate-std-dev 50 -a 6 -i 10 -I 20000 –microexon-search –min-segment-intron 10–max-segment-intron 50000)^44^. De novo RNA-seq assembly was performed using the Trinity pipeline with default options^45^. For RT-qPCR, total RNA was extracted from each stage of nematodes using TRI reagent (Sigma). cDNA was synthesized using iScript according to manufactures instruction (BioRad). RT-qPCR was performed using 1 μl cDNA and Power SYBR Green PCR Master Mix (Life technology) on the StepOnePlus System (Applied Biosystems).

To search for virus-like particles by electron microscopy, approximately 100,000 *B. xylophilus* individuals (strain Ka4, the original reference genome strain which was isolated in Ibaraki, Japan in 1994 and has been maintained by subculturing in FFPRI) were homogenized in 0.1 M sodium phosphate buffer at pH7.5. Cell debris was pelleted by low-speed centrifugation (8,000 g). The supernatant was adjusted to 6% PEG6000 and 0.125 M NaCl. After centrifugation at 10,000 g, the pellet was suspended in 0.03 M sodium phosphate buffer, pH7.5 and subjected to ultracentrifugation at 100,000 g. Extracts were negatively stained with 2% phosphotungstic acid (pH6.5) and examined in a Hitachi JEM2000EX transmission electron microscope at an 80 kV acceleration voltage.

LTRharvest^46^ was used to initially detect the presence of LTRs flanking the virus insertion (LTR lengths 10–1000 bp, total length 1–15 kbp, 90% minimum LTR similarity, TSD length 5–20 bp, seed 100 bp, looking for motif starting ‘tg’ and ending in ‘ca’). To assess the abundance of copies of the detected LTR retrotransposon in *the B. xylophilus* genome, *LTRharvest* was run with more sensitive parameters on the whole genome sequence (LTR lengths 100–1000 bp, total length 1–10 kbp, 85% minimum LTR similarity, TSD length 4–20 bp, seed 30 bp) to identify potential candidates in the genome. *LTRdigest*^47^ was used to subsequently annotate the candidates with protein domains from Pfam^48^ and GyDB^30^ as well as putative primer binding sites and polypurine tracts. The additional information was processed using custom scripts to remove low confidence candidates by only keeping (1) candidates with at least one protein domain, and (2) candidates with full LTR retrotransposon domain sets (reverse transcriptase, protease, integrase, RNAse H). The sequence of the nodavirus containing LTR element was then compared to the remaining genome-wide *LTRharvest* results as well as against the whole genome sequence using high sensitivity (E-value: 0.01) BLASTN and TBLASTX searches. Classification of the LTR element was by BLAST similarity search of the LTRs and *LTRharvest* protein matches against GyDB release 2.0, using the tool available at http://gydb.org/index.php/Blast.

To examine presence/absence of the eBxnv-1-containing element, genomic DNA reads from six *B. xylophilus* strains (Ka4C1, T4, S10-P3, S10-P9, C14-5 and OKD-F7)^31^ and *B. mucronatus* (Un1 strain) was mapped to the *B. xylophilus* v1.2 assembly with the gap-filled scaffold01281 using SMALT v0.7.4 (https://www.sanger.ac.uk/resources/software/smalt/) with options –x and –y 0.8. Variants in the scaffolds were called using the Picard tool (ver. 1.95) and GATK (version ver. 3.3.0) as described previously^31^. A maximum-likelihood tree was generated from SNP sites derived from 10 biggest scaffolds in the assembly using SNPhylo program^49^ with options (-m 0.1 –M 0.1 –l 0.9 –b 100). Presence of the element was confirmed by PCR amplification using genomic DNA and primers listed in Supplementary Table S2.

## Additional Information

**Accession Codes:** Nucleotide sequence of the eBxnv-1 region has been deposited to DDBJ/GenBank/EMBL under accession number LC158686. The authors have no competing interests to declare. B. xylophilus population sequence data, B. xylophilus RNA-seq data and DNA read data of B. mucronatus was deposited at DDBJ Sequence Read Archive (DRA) under BioProject numbers PRJDB3459, PRJDB3458 and PRJDB3460, respectively.

**How to cite this article**: Cotton, J. A. *et al*. An expressed, endogenous Nodavirus-like element captured by a retrotransposon in the genome of the plant parasitic nematode *Bursaphelenchus xylophilus. Sci. Rep.* **6**, 39749; doi: 10.1038/srep39749 (2016).

**Publisher's note:** Springer Nature remains neutral with regard to jurisdictional claims in published maps and institutional affiliations.

## Supplementary Material

Supplementary Information

## References

[b1] Bird A. F. & Bird J. The Structure of Nematodes. 2nd edn, 276–279 (Academic Press, 1991).

[b2] Foor W. E. Viruslike particles in a nematode. J Parasitol 58, 1065–1070 (1972). 4641873

[b3] Ibrahim I. K. A., Joshi M. M. & Hollis J. P. Swarming disease of nematodes: Host range and evidence for a cytoplasmic polyhedral virus in *Tylenchorhynchus martini*. Proc Helminthological Soc of Washington 45, 233–238 (1978).

[b4] Poinar G. O. Jr. & Hess R. Virus-like particles in the nematode Romanomermis culicivorax (Mermithidae). Nature 266, 256–257 (1977). 846569 10.1038/266256a0

[b5] Franz C. J., Zhao G., Felix M. A. & Wang D. Complete genome sequence of Le Blanc virus, a third Caenorhabditis nematode-infecting virus. Journal of virology 86, 11940, doi: 10.1128/JVI.02025-12 (2012). 23043172 PMC3486331

[b6] Felix M. A. . Natural and experimental infection of Caenorhabditis nematodes by novel viruses related to nodaviruses. PLoS biology 9, e1000586, doi: 10.1371/journal.pbio.1000586 (2011). 21283608 PMC3026760

[b7] Franz C. J. . Orsay, Santeuil and Le Blanc viruses primarily infect intestinal cells in Caenorhabditis nematodes. Virology 448, 255–264, doi: 10.1016/j.virol.2013.09.024 (2014). 24314656

[b8] Guo Y. R. . Crystal structure of a nematode-infecting virus. Proceedings of the National Academy of Sciences of the United States of America 111, 12781–12786, doi: 10.1073/pnas.1407122111 (2014). 25136116 PMC4156749

[b9] Jiang H. . Orsay virus utilizes ribosomal frameshifting to express a novel protein that is incorporated into virions. Virology 450–451, 213–221, doi: 10.1016/j.virol.2013.12.016 (2014). PMC3969245 24503084

[b10] Jiang H., Franz C. J. & Wang D. Engineering recombinant Orsay virus directly in the metazoan host Caenorhabditis elegans. Journal of virology 88, 11774–11781, doi: 10.1128/JVI.01630-14 (2014). 25078701 PMC4178717

[b11] Bekal S., Domier L. L., Niblack T. L. & Lambert K. N. Discovery and initial analysis of novel viral genomes in the soybean cyst nematode. The Journal of general virology 92, 1870–1879, doi: 10.1099/vir.0.030585-0 (2011). 21490246

[b12] Elsworth B., Wasmuth J. & Blaxter M. NEMBASE4: the nematode transcriptome resource. International journal for parasitology 41, 881–894, doi: 10.1016/j.ijpara.2011.03.009 (2011). 21550347

[b13] Brown D. J., Robertson W. M. & Trudgill D. L. Transmission of viruses by plant nematodes. Annu Rev Phytopathol 33, 223–249, doi: 10.1146/annurev.py.33.090195.001255 (1995). 18999960

[b14] Katzourakis A. & Gifford R. J. Endogenous viral elements in animal genomes. PLoS genetics 6, e1001191, doi: 10.1371/journal.pgen.1001191 (2010). 21124940 PMC2987831

[b15] Hayward A. & Katzourakis A. Endogenous retroviruses. Current biology: CB 25, R644–646, doi: 10.1016/j.cub.2015.05.041 (2015). 26241134

[b16] Crochu S. . Sequences of flavivirus-related RNA viruses persist in DNA form integrated in the genome of Aedes spp. mosquitoes. The Journal of general virology 85, 1971–1980, doi: 10.1099/vir.0.79850-0 (2004). 15218182

[b17] Holmes E. C. The evolution of endogenous viral elements. Cell host & microbe 10, 368–377, doi: 10.1016/j.chom.2011.09.002 (2011). 22018237 PMC7172163

[b18] Horie M. . Endogenous non-retroviral RNA virus elements in mammalian genomes. Nature 463, 84–87, doi: 10.1038/nature08695 (2010). 20054395 PMC2818285

[b19] Katzourakis A. Paleovirology: inferring viral evolution from host genome sequence data. Philosophical transactions of the Royal Society of London. Series B, Biological sciences 368, 20120493, doi: 10.1098/rstb.2012.0493 (2013). 23938747 PMC3758182

[b20] Henzy J. E. & Johnson W. E. Pushing the endogenous envelope. Philosophical transactions of the Royal Society of London. Series B, Biological sciences 368, 20120506, doi: 10.1098/rstb.2012.0506 (2013). 23938755 PMC3758190

[b21] Aiewsakun P. & Katzourakis A. Endogenous viruses: Connecting recent and ancient viral evolution. Virology 479–480, 26–37, doi: 10.1016/j.virol.2015.02.011 (2015). 25771486

[b22] Esnault C., Cornelis G., Heidmann O. & Heidmann T. Differential evolutionary fate of an ancestral primate endogenous retrovirus envelope gene, the EnvV syncytin, captured for a function in placentation. PLoS genetics 9, e1003400, doi: 10.1371/journal.pgen.1003400 (2013). 23555306 PMC3610889

[b23] Feschotte C. & Gilbert C. Endogenous viruses: insights into viral evolution and impact on host biology. Nature reviews. Genetics 13, 283–296, doi: 10.1038/nrg3199 (2012). 22421730

[b24] Kikuchi T. . Genomic insights into the origin of parasitism in the emerging plant pathogen Bursaphelenchus xylophilus. PLoS pathogens 7, e1002219, doi: 10.1371/journal.ppat.1002219 (2011). 21909270 PMC3164644

[b25] Jones J. T., Moens M., Mota M., Li H. & Kikuchi T. *Bursaphelenchus xylophilus*: opportunities in comparative genomics and molecular host-parasite interactions. Mol Plant Pathol. 9, 357–368 (2008). 18705876 10.1111/j.1364-3703.2007.00461.x PMC6640334

[b26] Sigrist C. J. . New and continuing developments at PROSITE. Nucleic acids research 41, D344–347, doi: 10.1093/nar/gks1067 (2013). 23161676 PMC3531220

[b27] Gough J., Karplus K., Hughey R. & Chothia C. Assignment of homology to genome sequences using a library of hidden Markov models that represent all proteins of known structure. J Mol Biol 313, 903–919, doi: 10.1006/jmbi.2001.5080 (2001). 11697912

[b28] Hansen J. L., Long A. M. & Schultz S. C. Structure of the RNA-dependent RNA polymerase of poliovirus. Structure 5, 1109–1122 (1997). 9309225 10.1016/s0969-2126(97)00261-x

[b29] Koonin E. V. The phylogeny of RNA-dependent RNA polymerases of positive-strand RNA viruses. The Journal of general virology 72 (Pt 9), 2197–2206, doi: 10.1099/0022-1317-72-9-2197 (1991). 1895057

[b30] Llorens C. . The Gypsy Database (GyDB) of mobile genetic elements: release 2.0. Nucleic acids research 39, D70–74, doi: 10.1093/nar/gkq1061 (2011). 21036865 PMC3013669

[b31] Palomares-Rius J. . Genome-wide variation in the pinewood nematode Bursaphelenchus xylophilus and its relationship with pathogenic traits. BMC Genomics 16, 845 (2015). 26493074 10.1186/s12864-015-2085-0 PMC4619224

[b32] Ball L. A. & Johnson K. L. In The Insect Viruses (eds L. K. Miller & L. A. Ball) 225–267 (Plenum Publishing Corporation, 1998).

[b33] Mori K. . Properties of a new virus belonging to nodaviridae found in larval striped jack (Pseudocaranx dentex) with nervous necrosis. Virology 187, 368–371 (1992). 1736540 10.1016/0042-6822(92)90329-n

[b34] Jatala P. Biological control of plant-parasitic nematodes. Annu Rev Phytopathol 24, 453–489 (1986).

[b35] Horie M. . An RNA-dependent RNA polymerase gene in bat genomes derived from an ancient negative-strand RNA virus. Sci Rep 6, 25873, doi: 10.1038/srep25873 (2016). 27174689 PMC4865735

[b36] Taylor D. J. & Bruenn J. The evolution of novel fungal genes from non-retroviral RNA viruses. BMC Biol 7, 88, doi: 10.1186/1741-7007-7-88 (2009). 20021636 PMC2805616

[b37] Geuking M. B. . Recombination of retrotransposon and exogenous RNA virus results in nonretroviral cDNA integration. Science 323, 393–396, doi: 10.1126/science.1167375 (2009). 19150848

[b38] Tanne E. & Sela I. Occurrence of a DNA sequence of a non-retro RNA virus in a host plant genome and its expression: evidence for recombination between viral and host RNAs. Virology 332, 614–622, doi: 10.1016/j.virol.2004.11.007 (2005). 15680426

[b39] Yook K. . WormBase 2012: more genomes, more data, new website. Nucleic acids research 40, D735–741, doi: 10.1093/nar/gkr954 (2012). 22067452 PMC3245152

[b40] Katoh K. & Toh H. Recent developments in the MAFFT multiple sequence alignment program. Brief Bioinform 9, 286–298, doi: 10.1093/Bib/Bbn013 (2008). 18372315

[b41] Capella-Gutierrez S., Silla-Martinez J. M. & Gabaldon T. trimAl: a tool for automated alignment trimming in large-scale phylogenetic analyses. Bioinformatics 25, 1972–1973, doi: 10.1093/Bioinformatics/Btp348 (2009). 19505945 PMC2712344

[b42] Stamatakis A. RAxML-VI-HPC: Maximum likelihood-based phylogenetic analyses with thousands of taxa and mixed models. Bioinformatics 22, 2688–2690, doi: 10.1093/Bioinformatics/Btl446 (2006). 16928733

[b43] Tsai I. J. . Transcriptional and morphological changes in the transition from mycetophagous to phytophagous phase in the plant-arasitic nematode Bursaphelenchus xylophilus. Mol. Plant Pathol. (2015). 10.1111/mpp.12261 PMC6638504 25831996

[b44] Kim D. . TopHat2: accurate alignment of transcriptomes in the presence of insertions, deletions and gene fusions. Genome Biol 14, R36, doi: 10.1186/gb-2013-14-4-r36 (2013). 23618408 PMC4053844

[b45] Grabherr M. G. . Full-length transcriptome assembly from RNA-Seq data without a reference genome. Nat Biotechnol 29, 644–652, doi: 10.1038/nbt.1883 (2011). 21572440 PMC3571712

[b46] Ellinghaus D., Kurtz S. & Willhoeft U. LTRharvest, an efficient and flexible software for de novo detection of LTR retrotransposons. BMC Bioinformatics 9, 18, doi: 10.1186/1471-2105-9-18 (2008). 18194517 PMC2253517

[b47] Steinbiss S., Willhoeft U., Gremme G. & Kurtz S. Fine-grained annotation and classification of de novo predicted LTR retrotransposons. Nucleic acids research 37, 7002–7013, doi: 10.1093/nar/gkp759 (2009). 19786494 PMC2790888

[b48] Finn R. D. . Pfam: the protein families database. Nucleic acids research 42, D222–230, doi: 10.1093/nar/gkt1223 (2014). 24288371 PMC3965110

[b49] Lee T. H., Guo H., Wang X., Kim C. & Paterson A. H. SNPhylo: a pipeline to construct a phylogenetic tree from huge SNP data. BMC Genomics 15, 162, doi: 10.1186/1471-2164-15-162 (2014). 24571581 PMC3945939

