# Supplementary material for: An expressed, endogenous Nodavirus-like element captured by a retrotransposon in the genome of the plant parasitic nematode Bursaphelenchus xylophilus
Source: Sci Rep. 2016 Dec 22;6:39749. doi: 10.1038/srep39749 (PMC5177903; doi:10.1038/srep39749)
Supplement: Supplementary Information [file srep39749-s1.pdf]

Supplementary Information

An expressed, endogenous Nodavirus-like element captured by a retrotransposon in the genome of the plant parasitic nematode *Bursaphelenchus xylophilus*

James A. Cotton[1], Sascha Steinbiss[1], Toshiro Yokoi[2], Isheng J. Tsai [1,3,4], Taisei Kikuchi [1,2,3*]

1 Wellcome Trust Sanger Institute, Wellcome Genome Campus, Hinxton, Cambridge, CB10 1SA, UK
2 Forestry and Forest Products Research Institute, Tsukuba 305-8687, Japan
3 Division of Parasitology, Faculty of Medicine, University of Miyazaki, Miyazaki 889-1692, Japan
4 Biodiversity Research Center, Academia Sinica, Taipei 11529, Taiwan

*Corresponding author
email: taisei_kikuchi@med.miyazaki-u.ac.jp, tel: +81-985850990, fax: +81-985843887

Supplementary Figures

Figure S1 – Region of multiple sequence alignment of nodavirus RdRp proteins, highlighting conservation of key domains across nodavirus proteins and the *B. xylophilus* element.
Figure S2 – Reverse transcriptase PCR, qPCR and RNA-seq evidence confirming expression of the gene in *B. xylophilus*.
Figure S3 – An example of transmission electron micrographs of *B. xylophilus* homogenate.

Supplementary Tables

Table S1 – Nodavirus RNA-dependent RNA polymerase sequences used in alignment and phylogenetic analysis
Table S2 – Primers used for eBxnv-1 genomic PCR, RT-PCR and sequencing

Supplementary Figure S1

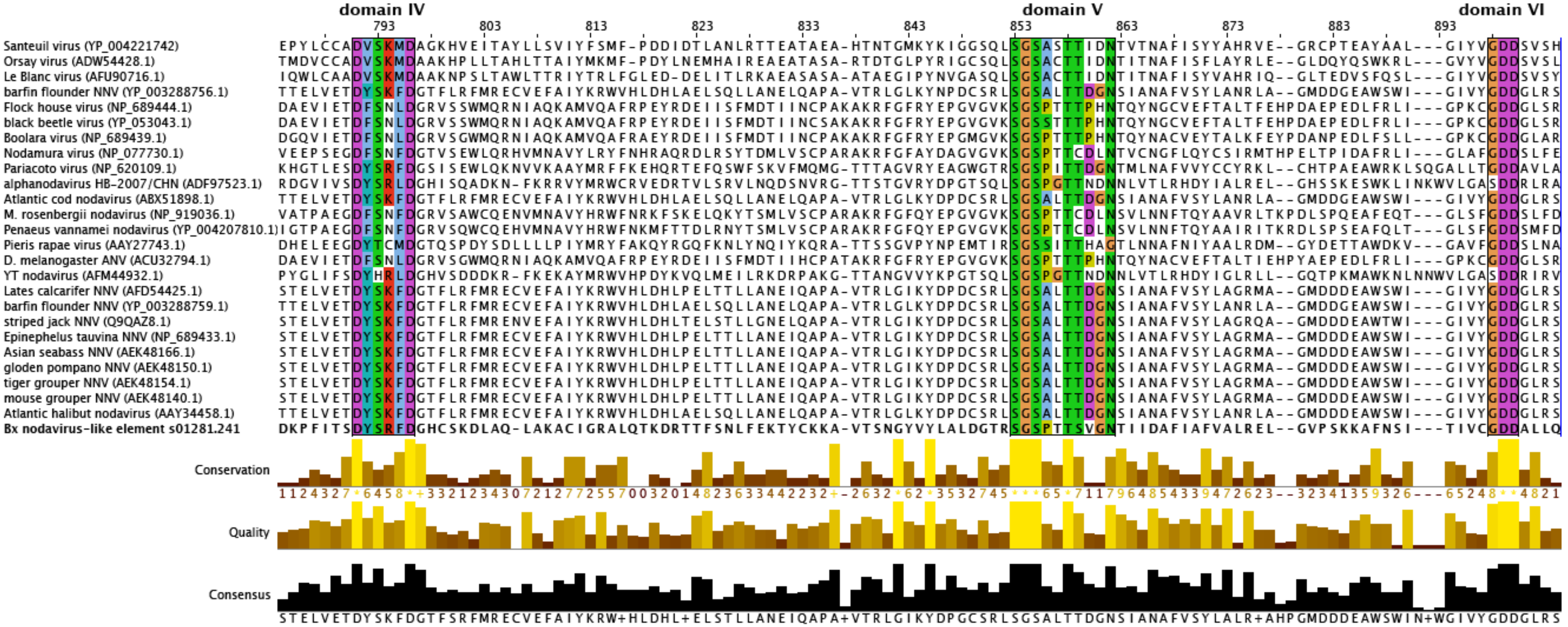

Region of multiple sequence alignment of nodavirus RdRp proteins, highlighting conservation of key domains across nodavirus proteins and the *B. xylophilus* element. Domains are numbered after Koonin [29]; note that the exact boundaries of 'conserved' domains are unclear and vary between authors, but highlighted blocks here include all highly-conserved residues from Prosite PS5057 (RdRp of positive ssRNA viruses catalytic domain).

Supplementary Figure S2

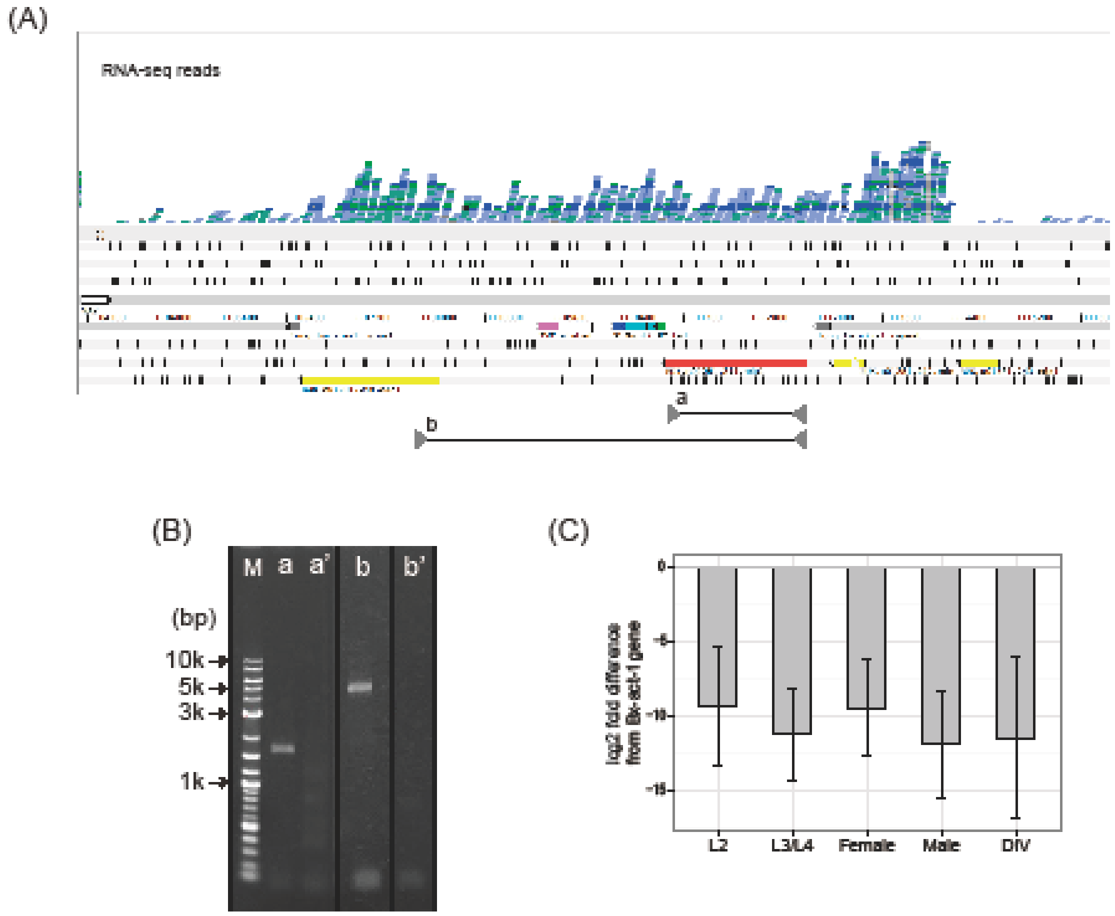

Reverse transcriptase PCR, qPCR and RNA-seq evidence confirming expression of the gene in *B. xylophilus*. (A) – A RNA-seq mapping view to the putative endogenised nodavirus RNA1 (eBxnv-1) locus in the Bursaphelenchus xylophilus genome and the surrounding LTR retrotransposon. (B) RT-PCR confirming expression of eBxnv-1 locus. Lower case alphabets (a and b) on each lane represent target regions shown in (A). Prime symbols (a' and b') represent genomic DNA contamination controls. M; 2-log ladder DNA size maker.(C) Relative expression levels of eBxnv-1 locus measured by quantitative polymerase chain reaction (qPCR). Expression of eBxnv-1 locus was compared with the actin gene (*Bx-act-1*) in multiple developmental stages of the nematodes. Error bars represent the standard deviations of the biological replicates. L2; second stage larva, L3/L4; thirds and forth stage larva, Female; adult females, Male; adult males, DIV; dauer (dispersal) forth stage larva.

Supplementary Figure S3

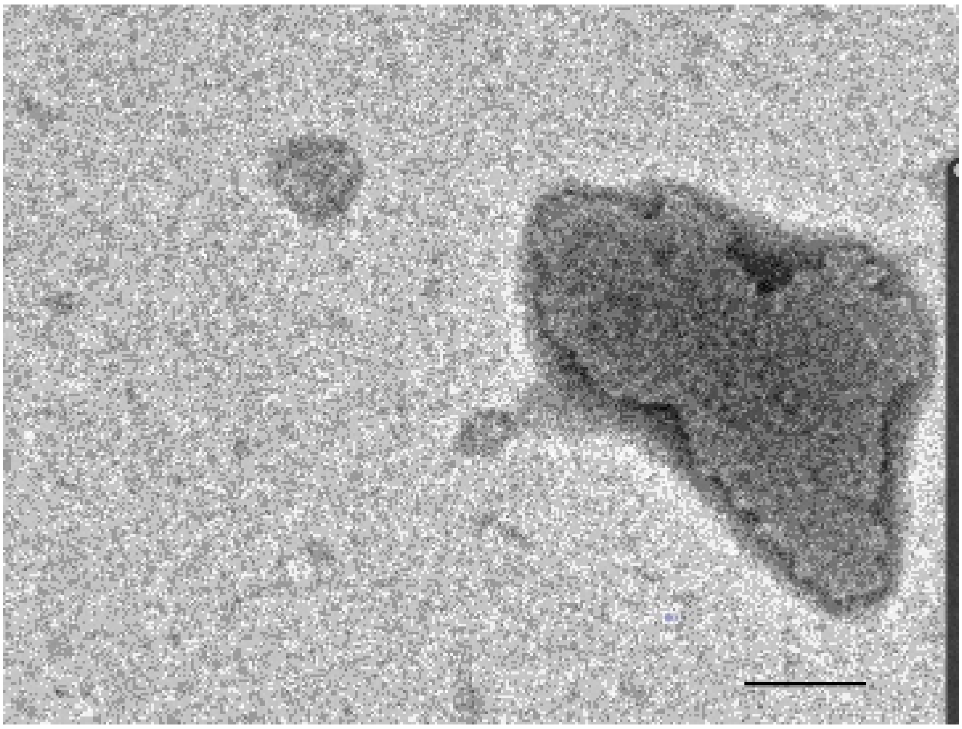

An example of transmission electron micrographs of *B. xylophilus* homogenate. No virus particle-like structures were observed in any field of view. The scale bars represent 100 nm.

Table S1 – Nodavirus RNA-dependent RNA polymerase sequences used in alignment and phylogenetic analysis.

Virus name	Genbank Accession Number	Taxonomy	Host taxonomic group	Host species	Virus isolation / discovery Reference	Virus sequenc e reference
Pariacoto virus	NP_620109	Alphanodavirus	insect	*Spodoptera eridania* (Southern armyworm)	(Zeddam, Rodriguez et al. 1999)	(Johnson, Zeddam et al. 2000)
Santeuil nodavirus	YP_004221742	Unclassified nodavirus	*nematode*	*Caenorhabditis briggsae*	*(Felix, Ashe et al. 2011)*	*(Felix, Ashe et al. 2011)*
Orsay nodavirus	ADW54428	Unclassified nodavirus	*nematode*	*Caenorhabditis elegans*	*(Felix, Ashe et al. 2011)*	*(Felix, Ashe et al. 2011)*
Le Blanc nodavirus	AFU90716	Unclassified nodavirus	*nematode*	*Caenorhabditis briggsae*	*(Franz, Zhao et al. 2012)*	*(Franz, Zhao et al. 2012)*
Nodamura virus	NP_077730	Alphanodavirus	Insect	Culex tritaeniorhynchus	(Scherer and Hurlbut 1967)	(Johnson, Price et al. 2003)
Macrobrachium rosenbergii nodavirus	NP_919036	Unclassified nodavirus	*crustacean*	*Macrobrachium rosenbergii* (giant freshwater prawn)	(Arcier, Herman et al. 1999)	(Sri Widada, Durand et al. 2003)
Penaeus vannamei nodavirus	YP_004207810	Unclassified nodavirus	crustacean	*Penaeus vannamei*	(Tang, Pantoja et al. 2007)	(Tang, Pantoja et al. 2011)
Drosophila melanogaster American nodavirus (ANV) SW-2009a	ACU32794	Unclassified nodavirus	Insect	*Drosophila melanogaster*	(Wu, Luo et al. 2010)	(Wu, Luo et al. 2010)

Flock house virus	NP_689444	Alphanodavirus	insect	*Costelytra zealandica* (grass grub)	(Dearing, Scotti et al. 1980)	(Johnson, Johnson et al. 2001)
black beetle virus	YP_053043	Alphanodavirus	insect	*Heteronycus arator*	(Longworth and Archibald 1975)	(Dasmahapatra, Dasgupta et al. 1985)
Boolarra virus	NP_689439	Alphanodavirus	insect	*Oncopera intricoides*	(Reinganum, Bashiruddin et al. 1985)	(Johnson, Johnson et al. 2001)
Tiger puffer nervous necrosis virus	YP_003288759	Betanodavirus	Fish	Takifugu rubripes (Tiger puffer)	(Iwamoto, Mori et al. 1999)	(Okinaka and Nakai 2008)
Barfin flounder nervous necrosis virus	YP_003288756	Betanodavirus	Fish	Barfin flounder	(Iwamoto, Mori et al. 1999)	(Okinaka and Nakai 2008)
Atlantic cod nodavirus	ABX51898	Betanodavirus	Fish	*Gadus morhua* (Atlantic Cod)	*(Nylund, Karlsbakk et al. 2008)*	*(Nylund, Karlsbakk et al. 2008)*
Atlantic halibut nodavirus	AAY34458	Betanodavirus	Fish	*Hippoglossus hippoglossus* (Atlantic halibut)	(Korsnes, Devold et al. 2005)	(Korsnes, Devold et al. 2005)
Mouse grouper Nervous Necrosis Virus	AEK48140	Betanodavirus	Fish	Mouse grouper	(Ransangan and Manin 2012)	(Ransangan and Manin 2012)
Lates calcarifer nervous necrosis virus	AFD54425	Betanodavirus	Fish	*Lates calcarifer*	(John, George et al. 2014)	unpublished
Pieris rapae virus	AAY27743	Unclassified nodavirus	insect	*Pieris rapae*	(Liu, Zhang et al. 2006)	(Liu, Zhang et al. 2006)

Alphanodavirus HB-2007/CHN	ADF97523	Alphanodavirus	insect	*Helicoverpa armigera*	(Bai, Wang et al. 2011)	(Bai, Wang et al. 2011)
YT nodavirus	AFM44932	Unclassified nodavirus	Unknown	Unknown-isolated from wastewater	unpublished	unpublished
striped jack nervous necrosis virus	Q9QAZ8	Betanodavirus	fish	striped jack	(Nagai and Nishizawa 1999)	(Nagai and Nishizawa 1999)
Asian seabass Nervous Necrosis Virus	AEK48166	Betanodavirus	Fish	Asian seabass	(Ransangan and Manin 2012)	(Ransangan and Manin 2012)
Epinephelus tauvina nervous necrosis virus	NP_689433	Betanodavirus	Fish	greasy grouper (Epinephelus tauvina)	(Chong, Ngoh et al. 1990)	(Tan, Huang et al. 2001)
Tiger grouper Nervous Necrosis Virus	AEK48154	Betanodavirus	Fish	Tiger grouper	(Ransangan and Manin 2012)	(Ransangan and Manin 2012)
Golden pompano Nervous Necrosis Virus	AEK48150	Betanodavirus	FIsh	Golden Pompano (*Trachinotus blochii*)	(Ransangan and Manin 2012)	(Ransangan and Manin 2012)

Additional References for Table S1

Arcier, J. M., F. Herman, D. V. Lightner, R. M. Redman, J. Mari and J. R. Bonami (1999). "A viral disease associated with mortalities in hatchery-reared postlarvae of the giant freshwater prawn Macrobrachium rosenbergii." Diseases of Aquatic Organisms **38**(3): 177-181.

Bai, H. M., Y. Wang, X. Li, H. T. Mao, Y. Li, S. L. Han, Z. L. Shi and X. W. Chen (2011). "Isolation and characterization of a novel alphanodavirus." Virology Journal **8**.

Chong, S. Y., G. H. Ngoh and M. Chew-Lim (1990). " Study of 3 tissue culture viral isolates from marine foodfish." Singapore Journal of Primary Industries **18**: 54-57.

Dasmahapatra, B., R. Dasgupta, A. Ghosh and P. Kaesberg (1985). "Structure of the black beetle virus genome and its functional implications." J Mol Biol **182**(2): 183-189.
Dearing, S. C., P. D. Scotti, P. J. Wigley and S. D. Dhana (1980). "A Small Rna Virus Isolated from the Grass Grub, Costelytra-Zealandica (Coleoptera, Scarabaeidae)." New Zealand Journal of Zoology **7**(2): 267-269.
Felix, M. A., A. Ashe, J. Piffaretti, G. Wu, I. Nuez, T. Belicard, Y. Jiang, G. Zhao, C. J. Franz, L. D. Goldstein, M. Sanroman, E. A. Miska and D. Wang (2011). "Natural and experimental infection of Caenorhabditis nematodes by novel viruses related to nodaviruses." PLoS Biol **9**(1): e1000586.
Franz, C. J., G. Zhao, M. A. Felix and D. Wang (2012). "Complete genome sequence of Le Blanc virus, a third Caenorhabditis nematode-infecting virus." J Virol **86**(21): 11940.
Iwamoto, T., K. Mori, M. Arimoto and T. Nakai (1999). "High permissivity of the fish cell line SSN-1 for piscine nodaviruses." Dis Aquat Organ **39**(1): 37-47.
John, K. R., M. R. George, B. Jeyatha, R. Saravanakumar, P. Sundar, K. P. Jithendran and E. O. Koppang (2014). "Isolation and characterization of Indian betanodavirus strain from infected farm-reared Asian seabass Lates calcarifer (Bloch, 1790) juveniles." Aquaculture Research **45**: 1481-1488.
Johnson, K. L., B. D. Price and L. A. Ball (2003). "Recovery of infectivity from cDNA clones of nodamura virus and identification of small nonstructural proteins." Virology **305**(2): 436-451.
Johnson, K. N., K. L. Johnson, R. Dasgupta, T. Gratsch and L. A. Ball (2001). "Comparisons among the larger genome segments of six nodaviruses and their encoded RNA replicases." J Gen Virol **82**(Pt 8): 1855-1866.
Johnson, K. N., J. L. Zeddam and L. A. Ball (2000). "Characterization and construction of functional cDNA clones of Pariacoto virus, the first Alphanodavirus isolated outside Australasia." J Virol **74**(11): 5123-5132.
Korsnes, K., M. Devold, A. H. Nerland and A. Nylund (2005). "Viral encephalopathy and retinopathy (VER) in Atlantic salmon Salmo salar after intraperitoneal challenge with a nodavirus from Atlantic halibut Hippoglossus hippoglossus." Dis Aquat Organ **68**(1): 7-15.
Liu, C., J. Zhang, F. Yi, J. Wang, X. Wang, H. Jiang, J. Xu and Y. Hu (2006). "Isolation and RNA1 nucleotide sequence determination of a new insect nodavirus from Pieris rapae larvae in Wuhan city, China." Virus Res **120**(1-2): 28-35.
Longworth, J. F. and R. D. Archibald (1975). "A virus of black beetle, Heteronychus arator (F.) (Coleoptera: Scarabaeidae)." New Zealand Journal of Zoology **2**: 233-236.
Nagai, T. and T. Nishizawa (1999). "Sequence of the non-structural protein gene encoded by RNA1 of striped jack nervous necrosis virus." J Gen Virol **80 (Pt 11)**: 3019-3022.
Nylund, A., E. Karlsbakk, S. Nylund, T. E. Isaksen, M. Karlsen, K. Korsnes, S. Handeland, R. Martinsen, T. Mork Pedersen and K. F. Ottem (2008). "New clade of betanodaviruses detected in wild and farmed cod (Gadus morhua) in Norway." Arch Virol **153**(3): 541-547.
Okinaka, Y. and T. Nakai (2008). "Comparisons among the complete genomes of four betanodavirus genotypes." Diseases of Aquatic Organisms **80**(2): 113-121.

Ransangan, J. and B. O. Manin (2012). "Genome analysis of Betanodavirus from cultured marine fish species in Malaysia." Vet Microbiol **156**(1-2): 16-44.
Reinganum, C., J. B. Bashiruddin and G. F. Cross (1985). "Boolarra Virus - a Member of the Nodaviridae Isolated from Oncopera-Intricoides (Lepidoptera, Hepialidae)." Intervirology **24**(1): 10-17.
Scherer, W. F. and H. S. Hurlbut (1967). "Nodamura virus from Japan: A new and unusual arbovirus resistant to diethyl ether and chloroform." Am. J. Epidemiol. **86**: 271-285.
Sri Widada, J., S. Durand, I. Cambournac, D. Qian, Z. Shi, E. Dejonghe, V. Richard and J. R. Bonami (2003). "Genome-based detection methods of Macrobrachium rosenbergii nodavirus, a pathogen of the giant freshwater prawn, Macrobrachium rosenbergii dot-blot, in situ hybridization and RT-PCR." J Fish Dis **26**(10): 583-590.
Tan, C., B. Huang, S. F. Chang, G. H. Ngoh, B. Munday, S. C. Chen and J. Kwang (2001). "Determination of the complete nucleotide sequences of RNA1 and RNA2 from greasy grouper (Epinephelus tauvina) nervous necrosis virus, Singapore strain." J Gen Virol **82**(Pt 3): 647-653.
Tang, K. F. J., C. R. Pantoja, R. M. Redman and D. V. Lightner (2007). "Development of in situ hybridization and RT-PCR assay for the detection of a nodavirus (PvNV) that causes muscle necrosis in Penaeus vannamei." Diseases of Aquatic Organisms **75**(3): 183-190.
Tang, K. F. J., C. R. Pantoja, R. M. Redman, S. A. Navarro and D. V. Lightner (2011). "Ultrastructural and sequence characterization of Penaeus vannamei nodavirus (PvNV) from Belize." Diseases of Aquatic Organisms **94**(3): 179-187.
Wu, Q. F., Y. J. Luo, R. Lu, N. Lau, E. C. Lai, W. X. Li and S. W. Ding (2010). "Virus discovery by deep sequencing and assembly of virus-derived small silencing RNAs." Proc Natl Acad Sci U S A **107**(4): 1606-1611.
Zeddam, J. L., J. L. Rodriguez, M. Ravallec and A. Lagnaoui (1999). "A noda-like virus isolated from the sweetpotato pest spodoptera eridania (Cramer) (Lep.; noctuidae)." J Invertebr Pathol **74**(3): 267-274.

Table S2 – Primers used for eBxnv genomic PCR, RT-PCR and sequencing

Primer ID	Sequence	Experiments
BUR.s01281.241-0f	ATGATTGATGTTGATTACTATGTCG	Figure 1(B)-a, b, c<br>Figure S1(B)-a, b
BUR.s01281.241-0r	TTACTGTGCCATCGCCGTCGTA	Figure 1(B)-d<br>Figure S1(B)-a<br>Sequencing
BUR.s01281.240-0r	CTAAGGAAGAAGAGATTCCGT	Figure 1(B)-a
BUR.s01281.240-909r	TACAATGTTCGTCCGGTTCA	Figure S1(B)-b<br>Sequencing
BUR.s01281.239-exon1-142r	CAGCCTTTTTCTTGGCTTTG	Figure 1(B)-b
BUR.s01281.239-exon2-59r	GCATCGTCTCCAAGCTTCTC	Figure 1(B)-c
BUR.s01281.243-7f	AATTTGGTCCTCTCATCATCAA	Figure 1(B)-d
BUR.s01281.241-1070f	CACGTCCAATAACGACGATG	Figure S1(C)<br>qPCR<br>Sequencing
BUR.s0281.241-1192r	CTTCCCTGGTGGACTTACCA	Figure S1(C)<br>qPCR<br>Sequencing
BUR.s01281.240-659f	ACGACGAGGAAACCATTGAC	Sequencing
s01281.240.1fRC	TTCGTTTTCCTTGGTACGG	Sequencing
s01281.240.0fRC	TTTCTCCATTTAAGAAGGTCAT	Sequencing
s01281.1108780-67f	TGGAAAGGACCGGAATTTCT	Sequencing
s01281.240.0r	CTAAGGAAGAAGAGATTCCGT	Sequencing
s01281.240.0rRC	TTTGACGGAATCTCTTCTTCCTTAG	Sequencing
241-2r	CAACTTCTCCTCCTATTTCTA	Sequencing
sXXXX68f	AAAGGGTAACTGAACCTCTA	Sequencing
241-00rRC	CTGACATTGCAAGTCGCGGAGCA	Sequencing

Table S3 - BLAST hits for *Bursaphelenchus xylophilus* gene models and retro-element related sequence regions. All tables show the top ten lowest e-value hits. (a-f) from BLASTp searches with proteins annotated in the B. xylophilus genome, against Genbank non-redundant protein sequences with default BLAST search parameters. (g) is a Blastx searches against gyDB 2.0 core protein database; (h) is a Blastn search against gyDB 2.0 LTR database.

(a) search with BUX_s01281.239

description	max score	total score	query coverage	e-value	identity	Accession
Brix domain-containing protein F44G4.1 [Toxocara canis]	430	430	62%	48e-147 6	62%	KHN82920.1
hypothetical protein CRE_01895 [Caenorhabditis remanei]	427	427	94%	6e-145	63%	XP_003117368.1
Brix domain containing protein [Haemonchus contortus]	424	424	93%	9e-145	62%	CDJ89331.1
Hypothetical protein CBG03232 [Caenorhabditis briggsae]	426	426	94%	2e-144	63%	XP_002631390.1
hypothetical protein CAEBREN_31495 [Caenorhabditis brenneri]	423	423	85%	2e-143	62%	EGT41930.1
brix domain-containing protein [Wuchereria bancrofti]	408	408	94%	4e-138	64%	EJW88320.1
Brix domain-containing protein F44G4.1 [Caenorhabditis elegans]	409	409	85%	7e-138	61%	NP_495918.1
hypothetical protein Y032_0001g65 [Ancylostoma ceylanicum]	403	403	85%	2e-136	66%	EYC34629.1

hypothetical protein Y032_0001g65 [Ancylostoma ceylanicum]	405	405	85%	3e-136	66%	EYC34631.1
Brix domain containing protein [Brugia malayi]	403	403	85%	4e-136	63%	XP_001900144.1

(b) search with BUX_s01281.240

description	max score	total score	query coverage	e-value	identity	Accession
hypothetical protein ZOSMA_11347G00010 [Zostera marina]	108	108	28%	3e-22	35%	KMZ75581.1
Hypothetical protein CBG24593 [Caenorhabditis briggsae]	90.1	90.1	22%	2e-15	31%	XP_002648367.1
Protein CBG24593 [Caenorhabditis briggsae]	90.1	90.1	22%	2e-15	31%	CAP21162.2
Hypothetical protein CBG23765 [Caenorhabditis briggsae]	89.7	89.7	22%	2e-15	31%	XP_002647899.1
hypothetical protein CRE_21848 [Caenorhabditis remanei]	89.7	136	70%	3e-15	30%	XP_003100218.1
Protein CBG23765 [Caenorhabditis briggsae]	89.7	89.7	22%	3e-15	31%	CAP20531.2
Hypothetical protein CBG24837 [Caenorhabditis briggsae]	89.7	89.7	22%	3e-15	31%	XP_002648539.1
Protein CBG24837 [Caenorhabditis briggsae]	89.4	89.4	22%	3e-15	31%	CAP21363.2
Hypothetical protein CBG19856 [Caenorhabditis briggsae]	89.0	89.0	22%	4e-15	30%	XP_002633824.1
Protein CBG19855 [Caenorhabditis briggsae]	89.0	150	22%	6e-15	30%	CAP37031.2

(c) search with BUX_s01281.241

description	max score	total score	query coverage	e-value	identity	Accession
RNA-dependent RNA polymerase (RdRp) [Bursaphelenchus xylophilus]	1187	1187	100%	0.0	100%	BAV60982.1
protein A [Pariacoto virus]	330	330	98%	6e99	36%	NPfl620109.1
RNAdependent RNA polymerase [Alphanodavirus HB2007/ CHN]	330	330	99%	1e98	35%	ADF97523.1
protein A [Mosinovirus]	313	313	98%	1e92	33%	AIO11151.1
RecName: Full=RNAdirected RNA polymerase; Short=RdRp; AltName: Full=RNA replicase	315	315	99%	1e92	33%	Q3KSM3.1
RNAdependent RNA polymerase [YT nodavirus]	281	281	94%	3e84	33%	AFM44932.1
putative RdRp [Bat guano associated nodavirus GF4n]	265	265	99%	7e75	32%	ADI48250Æ1
putative polymerase [Craigmillar Park virus]	251	251	99%	5e70	31%	AMO03241Æ1
hypothetical protein [Craigies Hill virus]	243	243	99%	1e67	31%	AKH67440Æ1
putative replicase [Craigies Hill virus]	244	244	99%	1e67	31%	AKH40302Æ1

(d) search with BUX_s01281.242

**description**	**max score**	**total score**	**query coverage**	**e-value**	**identity**	**Accession**
hypothetical protein CAEBREN_32237 [Caenorhabditis brenneri]	36.6	36.6	70%	2.0	31%	EGT51229.1

(e) search with BUX_s01281.243

**description**	**max scor e**	**total scor e**	**query coverag e**	**e-valu e**	**identit y**	**Accession**
metal-dependent phosphohydrolase [Nocardiopsis sp. RV163]	39.7	39.7	46%	0.59	37%	WP_047868278. 1
retrotransposon protein, putative, unclassified [Oryza sativa Japonica Group]	37.7	37.7	50%	3.7	24%	ABF94845.1
Os08g0451600 [Oryza sativa Japonica Group]	37.4	37.4	50%	5.1	27%	NP_001061948.2
metal-dependent phosphohydrolase [Nocardiopsis dassonvillei]	36.6	36.6	68%	6.8	31%	WP_013151834. 1

hypothetical protein [Streptomyces sp. NRRL S-646]	35.0	35.0	28%	8.2	41%	WP_030936029.1
gamma-glutamyltranspeptidase [Marinobacter excellens]	36.6	36.6	59%	8.8	36%	WP_044389523.1
Sulfate Permease (SulP) Family [Phytophthora infestans T30-4]	36.6	36.6	43%	9.7	32%	XP_002895548.1

(f) search with BUX_s01281.244

**description**	**max score**	**total score**	**query coverage**	**e-value**	**identity**	**Accession**
membrane protein [Snodgrassella alvi]	38.1	38.1	61%	5.8	24%	WP_037489241.1

(g) search with region scaffold01281:1108824-1109142; annotated as reverse transcriptase by LTRdigest

**Subject mapping**	**Score**	**e-value**	**Similarity**
RNaseH_Mabel	127	1.18E-11	64.22
RNaseH_Kobel	121	1.02E-10	56.48
RNaseH_Zebel	117	3.94E-10	55.66
RNaseH_Tamy	113	1.47E-09	57.41
RNaseH_Spirobel	104	5.98E-08	53.27
RNaseH_Roo	94	8.08E-07	52.38
RNaseH_Hydra3-1	89	7.21E-06	52.34
RNaseH_Saci-6	78	6.18E-05	55.36
RNaseH_Tribel	75	0.000632933	48.18
RNaseH_Cubel	70	0.0022579	46.15

(h) search with region scaffold01281:1106433-1106565; annotated as the 5' long terminal repeat unit by LTRdigest

**Subject mapping**	**Score**	**e-value**	**Similarity**
LTR5'_GypsySL_04	13	0.413506	100
LTR5'_CopiaSL_monotypic|Chr02_2s24	13	0.413506	94.12
LTR3'_Ulysses	13	0.413506	100
LTR5'_Ulysses	13	0.413506	100
LTR5'_GypsySL_monotypic|Chr08_2s54	12	1.63391	100
LTR5'_GypsySL_monotypic|Chr08_2s54	12	1.63391	100
LTR5'_GypsySL_monotypic|Chr08_2s54	11	6.45621	100
LTR5'_GypsySL_monotypic|Chr08_2s54	11	6.45621	100
LTR5'_GypsySL_07	12	1.63391	93.75

LTR5'_GypsySL_07	11	6.45621	100
